# Genetic evaluation of mastitis liability and recovery through longitudinal analysis of transition probabilities

**DOI:** 10.1186/1297-9686-44-10

**Published:** 2012-04-04

**Authors:** Jessica Franzén, Daniel Thorburn, Jorge I Urioste, Erling Strandberg

**Affiliations:** 1 Department of Animal Breeding and Genetics, Swedish University of Agricultural Sciences, Box 7023, Uppsala 750 07, Sweden; 2 Department of Statistics, Stockholm University, Stockholm 106 91, Sweden; 3 Departamento de Producción Animal y Pasturas, Facultad de Agronomía, UDELAR, Garzón 780, Montevideo 12900, Uruguay

## Abstract

**Background:**

Many methods for the genetic analysis of mastitis use a cross-sectional approach, which omits information on, e.g., repeated mastitis cases during lactation, somatic cell count fluctuations, and recovery process. Acknowledging the dynamic behavior of mastitis during lactation and taking into account that there is more than one binary response variable to consider, can enhance the genetic evaluation of mastitis.

**Methods:**

Genetic evaluation of mastitis was carried out by modeling the dynamic nature of somatic cell count (SCC) within the lactation. The SCC patterns were captured by modeling transition probabilities between assumed states of mastitis and non-mastitis. A widely dispersed SCC pattern generates high transition probabilities between states and vice versa. This method can model transitions to and from states of infection simultaneously, i.e. both the mastitis liability and the recovery process are considered. A multilevel discrete time survival model was applied to estimate breeding values on simulated data with different dataset sizes, mastitis frequencies, and genetic correlations.

**Results:**

Correlations between estimated and simulated breeding values showed that the estimated accuracies for mastitis liability were similar to those from previously tested methods that used data of confirmed mastitis cases, while our results were based on SCC as an indicator of mastitis. In addition, unlike the other methods, our method also generates breeding values for the recovery process.

**Conclusions:**

The developed method provides an effective tool for the genetic evaluation of mastitis when considering the whole disease course and will contribute to improving the genetic evaluation of udder health.

## Background

Mastitis is a common disease in dairy cattle with severe economic consequences [1]. It has been shown that susceptibility to the disease varies between breeds and individuals, with heritabilities ranging from 0.07 to 0.12 [2,3]. Genetic evaluation of the disease is an important issue and a wide range of methods is available. Methods can be divided into cross-sectional or longitudinal approaches. Cross-sectional methods consider each lactation as a static process, whereas longitudinal methods model changes in disease states during the lactation. The variables mostly used in mastitis analyses are recorded cases of clinical mastitis (CM) or somatic cell counts (SCC). Routine recording of CM is not performed in most countries and SCC can be used as a proxy in the genetic evaluation, due to its high genetic correlation with CM [4-6].

Cross-sectional analyses of recorded cases of CM consider either CM as a binary variable, distinguishing between absence of cases and occurrence of at least one case during lactation, or the lactation average SCC. A shortcoming of these methods is that they ignore the dynamic nature of mastitis, e.g., multiple cases of mastitis or the longitudinal SCC pattern. The dynamic nature of mastitis is taken into account to various extents in longitudinal approaches, for example by counting the number of CM cases during a lactation using a Poisson model [7] or by modeling presence or absence of CM in pre-specified lactation intervals using a longitudinal threshold liability model, which gives as many heritability estimates as intervals [8,9]. Other longitudinal methods include time in lactation either as a variable in the model, e.g., time to first case in a survival analysis [10], or as an explanatory variable in a random regression model, making heritability a function of time [11-14].

In [15-17], SCC was analyzed longitudinally, using different patterns based on deviations from the typical SCC curve, to investigate how these patterns might be related to pathogen-specific cases of CM. Traits derived from lactation SCC data have also been used to capture longitudinal features such as mastitis intensity through the number of peaks (consecutive SCC test days above a certain level), recovery pace through peak lengths, and lactation fluctuation by the standard deviation of SCC [18-20].

In longitudinal studies, the degree at which the dynamic nature of mastitis is accommodated varies, but research in this field is moving to more sophisticated methods in which the longitudinal approach is one aspect that is stressed. Results from such studies show that considering the longitudinal aspect improves the genetic evaluation of CM.

We present an alternative longitudinal approach in which genetic evaluation of mastitis is performed based on changes in SCC during lactation. These changes are captured by modeling transition probabilities between assumed states of mastitis and non-mastitis. The method simultaneously models the transition probability of developing mastitis and the probability of recovering from an infection. The former has been the focus of many studies but the infection recovery process has been little investigated. In our approach, we model both aspects to capture as much genetic information as possible from the SCC lactation pattern.

## Methods

### Transition probability model

We assumed that during lactation a cow can move between two possible states: mastitis and non-mastitis, referred to as diseased (D) and healthy (H) states. A pre-specified SCC level marks the boundary between the states. The boundary B(τ) varies along the lactation according to a multiple *m *of an average lactation curve L(τ) for primiparous cows, according to

(1) $$B\left(\tau \right)=m\times L\left(\tau \right)$$

where  is time in lactation, starting at calving. L(τ) was modeled by a spline function, parameterized according to data in the Jälla research herd, University of Agricultural Sciences, Uppsala, Sweden [21]. Two different values of *m *were used (*m = 10 *and *15*). The probability of moving from a healthy to a diseased state for cow *i *is denoted $\pi_{i}^{\left(HD\right)}$ and the probability of moving from a diseased to a healthy state, $\pi_{i}^{\left(DH\right)}$. The transition probabilities for cow *i *may be summarized in a transition matrix *Tr_i_*,

(2) $$Tr_{i}=\left[\begin{array}{cc} {\pi_{i}}^{\left(HD\right)} & 1-{\pi_{i}}^{\left(HD\right)}\\ 1-{\pi_{i}}^{\left(DH\right)} & {\pi_{i}}^{\left(DH\right)} \end{array}\right]$$

which gives the probabilities of changing states or remaining in the current state. A transition between two particular states is repeatable, i.e. a cow can be in a specific state more than once during a given lactation. An episode is defined as the duration of each state; after a transition, a new episode begins, leading to multiple episodes within the lactation. The transition probabilities reflect the SCC fluctuations. A widely dispersed pattern, i.e. with many fluctuations between high and low SCC levels, will generate higher values of $\pi_{i}^{\left(HD\right)}$ and $\pi_{i}^{\left(DH\right)}$ (high transition probabilities between states) and lower values of $1-{\pi_{i}}^{\left(HD\right)}$ and $1-{\pi_{i}}^{\left(DH\right)}$ (low probabilities of remaining in a current state), compared to values for an individual with a SCC pattern similar to the average lactation curve. A desired structure of the transition matrix would be to have high values of $1-{\pi_{i}}^{\left(HD\right)}$ and $\pi_{i}^{\left(DH\right)}$ (obviously together with low values of $\pi_{i}^{\left(HD\right)}$ and $1-{\pi_{i}}^{\left(DH\right)}$), which reflect an individual who rarely develops mastitis but if it does, has a quick recovery.

When modeling survival data with repeated events, such as repeated mastitis cases during lactation, multilevel models are effective and powerful tools [22]. In these models, repeated transitions are viewed as an extra level in a higher level hierarchical structure. Here, we use a three-level structure, in which episodes are nested within cows, and herds and sires are cross-classified on the highest level. Data for mastitis and SCC are most often interval-censored, i.e. the actual time for a transition between states is unknown. When data is collected retrospectively, a state change is only known to have occurred at some point between two data collection times. Although the underlying process is continuous, the structure of the data calls for a model that recognizes its discrete nature. Therefore, a continuous time survival model such as the Cox proportional hazards model [23] was rejected in favor of a multilevel discrete time survival model. Statistical descriptions and evaluations of multilevel survival models with repeated events can be found in [24-26]. By restructuring and expanding the dataset, the multilevel binary response model can be fitted using logistic regression, as in our study, or using other standard methods for discrete response data.

The transition probability *π_ijk _*is the discrete equivalent of the continuous time hazard function and is defined as the probability that a transition occurs at some time between any two measurements for cow *i*, daughter of sire *j*, and member of herd *k*. The model for the transition probability of moving from a healthy to a diseased state, $\pi_{ijk}^{\left(HD\right)}$, is expressed as:

(3) $$\begin{array}{c} f_{ijkt}^{\left(HD\right)}\sim Ber\left(\pi_{ijk}^{\left(HD\right)}\right)\text{and}\\ \text{logit}\left(\pi_{ijk}^{\left(HD\right)}\right)=\beta^{\left(HD\right)}+s_{j}^{\left(HD\right)}+h_{k}^{\left(HD\right)}+e_{ijk}^{\left(HD\right)}\; \end{array}$$

where $f_{ijkt}^{\left(HD\right)}$ = 1 if a transition occurred in time interval *t *and otherwise = 0. A more complete description of the binary variable $f_{ijkt}^{\left(HD\right)}$ and the definition of *t *are given in the "Data expansion" section. Variables $s_{j}^{\left(HD\right)}\sim N\left(0,{\left(\sigma_{S}^{\left(HD\right)}\right)}^{2}\right)$ and $h_{k}^{\left(HD\right)}\sim N\left(0,{\left(\sigma_{h}^{\left(HD\right)}\right)}^{2}\right)$ are random sire and herd effects and $e_{ijk}^{\left(HD\right)}\sim N\left(0,{\left(\sigma_{e}^{\left(HD\right)}\right)}^{2}\right)$ is the random residual effect. The transition probability of recovery $\pi_{ijk}^{\left(DH\right)}$, i.e. the probability of moving from a diseased to healthy state, is modeled in a corresponding way,

(4) $$\begin{array}{c} f_{ijkt}^{\left(DH\right)}\sim Ber\left(\pi_{ijk}^{\left(DH\right)}\right)\text{and}\\ \text{logit}\left(\pi_{ijk}^{\left(DH\right)}\right)=\beta^{\left(DH\right)}+s_{j}^{\left(DH\right)}+h_{k}^{\left(DH\right)}+e_{ijk}^{\left(DH\right)}\; \end{array}$$

with random effects $s_{j}^{\left(DH\right)}\sim N\left(0,{\left(\sigma_{S}^{\left(DH\right)}\right)}^{2}\right)$, $h_{k}^{\left(DH\right)}\sim N\left(0,{\left(\sigma_{h}^{\left(DH\right)}\right)}^{2}\right)$ and $e_{ijk}^{\left(DH\right)}\sim N\left(0,{\left(\sigma_{e}^{\left(DH\right)}\right)}^{2}\right).$

The two transitions probabilities are expressed conditional on the current state. In a given interval, a cow can only change states in one direction (or stay in the same state).

### Simulations and Bayesian inference

As shown by Allison [27] and Browne et al. [22], the likelihood function for a discrete time multilevel model is equivalent to the likelihood for the regression analysis of a dichotomous dependent variable. Therefore, the multilevel discrete time model can be fitted using standard software packages for logistic regression, where the response variable is the binary indicator of the occurrence of an event $f_{ijkt}^{\left(HD\right)}$ or $f_{ijkt}^{\left(DH\right)}$. The choice of inference of the model is open to both a classical or a Bayesian estimation approach, however, the modular nature of Markov chain Monte Carlo (MCMC) algorithms in Bayesian inference make them an attractive choice to estimate discrete time repeated events [22]. In addition, it has been shown that Bayesian estimates are less biased than maximum likelihood estimates for random-effect logistic regression models [28]. In this paper, Bayesian estimates were obtained with the multilevel software program MLwiN, which is developed by the Centre of Multilevel Modeling, University of Bristol [29]. This software offers the choice of classical or Bayesian estimation and has many features and options to fit multilevel models, including parameter expansion, which is discussed in the next section. The MCMC approach in MLwiN consists of iterative Metropolis-Hastings (MH) and Gibbs sampler steps to successively update the model parameters. Gibbs sampling is used for variances and univariate-update random-walk Metropolis sampling with Gaussian proposal distributions for fixed effects and residuals. For a complete description of the MCMC steps and conditional densities, see [22,30].

Vague priors were used. The variances of the random effects had an inverse Gamma prior with small parameters and a uniform prior was used for the fixed intercepts (*β*):

$$\begin{array}{c} p\left(\sigma^{2}\right)\sim {\text{Γ}}^{-1}\left(\varepsilon ,\varepsilon \right)\\ p\left(\beta \right)\propto 1\\ \varepsilon =0.001. \end{array}$$

Estimates of parameters in the HD direction were based on 10 000 iterations with a burn in of 500, whereas estimates in the DH direction were based on 100 000 iterations with a burn in of 5000. The strongly reduced dataset for DH estimations called for longer iteration chains to obtain convergence and to estimate variances with the same accuracies as the HD estimates.

### Parameter expansion

As previously mentioned, MCMC methods can reduce estimation bias in the discrete time survival model. However, data expansion results in very large datasets for which the MCMC algorithm can be slow and generate chains that exhibit poor mixing. This was apparent for the MCMC algorithms of *σ_e_*, especially for estimation of parameters in the DH direction. One of the main causes of poor mixing in the MCMC algorithm is correlated model parameters. When correlations exist between random effects and their variances, and the variances are close to zero, the MCMC chain can get stuck close to zero, both for the variance and its random effect. Parameter expansion is a reparameterization method, which reformulates the statistical model by replacing certain parameters with others that are not correlated and therefore generate MCMC chains with much better mixing than the original chains [22,31,32]. The reparameterization is done in such a way that it is possible to recover the original parameters in the model. The method is a built-in option in MLwiN and is used in the estimations reported in this paper, resulting in much better mixing and therefore faster convergence and less need for long iteration chains.

### Simulated data

The process used to generate data on mastitis was an extension of the simulations by Carlén et al. [33] and Schneider et al. [34]. These studies generated data on milk production, interval between calving and first ovulation, conception and mastitis liability. Mastitis history data were simulated as a binary trait until the first case of mastitis or the end of the lactation. For this study, we extended the program to include repeated cases of mastitis and also SCC data connected to every binary response.

Weekly SCC values were simulated for 12 datasets that consisted of all possible combinations of two population sizes, two mastitis frequencies and three genetic correlations between mastitis and recovery liabilities. Five replicates were used for each population structure. Two population sizes were used i.e. 24 000 and 60 000 first-parity cows. The cows were the daughters of 400 unrelated sires distributed over 1200 herds, with a fixed herd size of 20 or 50, resulting in average daughter group sizes of 60 and 150, respectively. The larger population size is similar in structure to the situation in Sweden, where the average size of herds participating in official milk recording varied between 50 and 60 in the past five years [35], and the number of daughters per young bull tested for mastitis resistance ranged from 150 to 200 [36]. Without prior knowledge about the possible genetic correlation between mastitis and recovery liabilities, three correlations were simulated: -0.2, 0, and 0.2. Furthermore, two mastitis incidence scenarios were used (Table 1). Scenario one was chosen to reflect previous estimates of the incidence of mastitis in field data of Swedish first-parity cows [5,11,33], where a case of mastitis was defined as a veterinary-treated clinical mastitis. The recurrence rate of CM was based on data from the research herd of the Department of Animal Breeding and Genetics, Swedish University of Agricultural Sciences [1]. The second scenario was not justified by previous studies but was chosen to take into consideration the possible higher frequencies of mastitis in multiparous cows and the fact that not all cases of mastitis may be veterinary-treated. It is also of theoretical interest to investigate the difference in performance of the method for different frequencies since it may be useful for other repeated disease data with higher frequencies. For both scenarios, the average probability of recovery from one week to the next, was 0.65 (equal to the rate in the Jälla research herd [21]). If a cow develops mastitis in a given week, the probability that it is still infected the following week is 0.35, 0.35^2 ^= 0.123 for two weeks later, 0.35^3 ^= 0.043 for three weeks later, etc..

**Table 1:** Average values for the two mastitis frequency scenarios

	Scenario 1	Scenario 2
1. Mastitis lactation (%)	16.5	40.0
2. Mastitis cases per lactation	0.28	0.95
3. Mastitis cases per affected lactation	1.50	2.40

1. Percent of lactations with at least one case of clinical mastitis; 2. Total number of cases/total number of lactations; 3. Total number of cases/total number of lactations with at least one case of clinical mastitis

Mastitis history data were simulated as a binary trait with underlying normally distributed mastitis and recovery liabilities. The mastitis and recovery liabilities for each cow were modeled by a herd effect, the animal's breeding value, and an environmental value. Breeding values for the cows were simulated by adding half the breeding value of the sire, half the breeding value of the dam and a Mendelian sampling term. The environmental value corresponds to a permanent cow effect, which means that the liability value represents a mean liability over lactation. A weekly random variable was added to allow the mastitis liability to vary from week to week. Before the weekly variations were added, the variance in the liabilities (~*N*(0, 0.8^2^)) was chosen to give a mastitis heritability of 0.1, which is in line with what is normally found in cross-sectional linear models (on the underlying scale) [5,37,38]. Together with the weekly random component (~*N*(0, 0.6^2^)), variances added up to a phenotypic variance of 1. The additive genetic variance was 0.036, which resulted in a simulated heritability of 0.036 for the weekly data (0.039 if the herd variance of 0.072 is excluded from the phenotypic variance). The recovery liabilities were simulated in the same way and with the same parameters.

For cases in which mastitis did not develop in the previous week, the mastitis liability of the current week generated the next binary outcome. If the resulting mastitis liability was above a defined threshold which corresponded to the targeted mastitis frequency, the cow developed mastitis. If mastitis did develop in the previous week, the current recovery liability decided the next week's binary outcome. If the recovery liability was above the threshold of -0.4 (corresponding to the relative recovery frequency of 0.65), the cow was free from the infection, and if not, the cow remained infected for (at least) another week.

The binary CM data was then used to simulate SCC observations: values for uninfected test days (days when the cow's SCC level is measured) were simulated as random deviations (*σ *= 0.64 according to previous studies [1]) from a baseline curve and test days with mastitis infections as random deviations from a function with instant SCC increase, followed by a successive decline down to the baseline level. The baseline curve was modeled by a three-phase linear spline function parameterized according to [39]. Another spline function expressed the immediate increase of SCC from the baseline at the time of infection and the successive decline down to the baseline level during the following weeks. This spline function was created according to findings by De Haas et al. [15] by generalizing the effects of *Staphylococcus aureus, Escherichia coli, Streptococcus dysgalactiae*, and *Streptococcus uberis *on SCC.

### Data expansion

Before applying the method to the data, the sequence of SCC lactation data was converted into a sequence of binary responses, which indicated whether a transition had occurred within each time interval. Cow, sire, and herd indicators were repeated as many times as there were binary responses in the lactation. Furthermore, a time indicator was added, to number the time intervals repeatedly until a transition. After a transition, the counter started over again and ran until the next transition or the end of the lactation. There was no restriction on the cows having the same number of measurements or the same interval between measurements. In theory, a cow only needs two consecutive measurements although, in practice, very short series carry little information. Long intervals between measurements can also lead to information loss due to missed transitions. However, evaluation concerns the performance of the sires, not that of the individual cows, thus longer intervals between measurements can to some extent be compensated by more daughters per sire.

The *t*:th SCC value (in order within a lactation) for cow *i*, daughter of sire *j*, and member of herd *k *is denoted y*_ijkt_, t *= 1,..., *T_i _*where *T_i _*is the number of measurements for cow *i *within the same lactation. The discrete variable *t *should not be confused with  which is a continuous variable for time in lactation, starting at calving. The binary response *h_ijkt _*states whether the *t*:th order SCC value for cow *i *is below (H) or above (D) the boundary stated in (1) and is formally expressed as

(6) $$h_{ijkt}=\left\{\begin{array}{c} 1\text{ if }{\text{y}}_{ijkt}>B\left(\tau_{t}\right)\\ \text{0 if }{\text{y}}_{ijkt}\leq B\left(\tau_{t}\right) \end{array}\right)$$

where $\tau_{t}$ is the time since calving for the *t*:th order response of cow *i*.

Two new datasets were created out of the binary sequence *h_ijkt_, t *= 1,..., *T_i_*. The first dataset was used to analyze mastitis liability and contained transitions from healthy to diseased states. The second dataset contained transitions from diseased to healthy states and was used to analyze the recovery process. The first dataset was much larger than the second because it included all cows, while the second contained only cows that had developed mastitis at least once. Transitions between states were recorded as a binary variable *f_ijkt_*, which states whether or not a transition took place between two consecutive measurements, *t *and *t *+ 1. For each cow and lactation, two binary series were created according to

(7) $$f_{ijkt}^{\left(HD\right)}=\left\{\begin{array}{c} 1\;\text{if}\;{\text{h}}_{ijkt}=0\;\text{and}\;{\text{h}}_{ijk\left(t+1\right)}=1\\ \text{0}\;\text{if}\;{\text{h}}_{ijkt}=0\;\text{and}\;{\text{h}}_{ijk\left(t+1\right)}=0\\ \text{delete}\;\text{if}\;{\text{h}}_{ijkt}=1\;\text{and}\;{\text{h}}_{ijk\left(t+1\right)}\text{ = 0}\;\text{or}\;1 \end{array}\right)$$

for H to D transitions and

(8) $$f_{ijkt}^{\left(DH\right)}=\left\{\begin{array}{c} 1\text{ if }{\text{h}}_{ijkt}=1\text{ and }{\text{h}}_{ijk\left(t+1\right)}=0\\ \text{0 if }{\text{h}}_{ijkt}=1\text{ and }{\text{h}}_{ijk\left(t+1\right)}=1\\ \text{delete if }{\text{h}}_{ijkt}=0\text{ and }{\text{h}}_{ijk\left(t+1\right)}=0\text{ or 1} \end{array}\right)$$

for D to H transitions (t = 1,..., *T_i_*-1). For example, a sequence of **h***_ijk _*= [0010001110100], containing 13 measurements and three cases of classified mastitis (one which lasted over three measurements) for cow *i*, generated $f_{ijk}^{\left(HD\right)}=\left[0100110\right]$ and $f_{ijk}^{\left(DH\right)}=\left[10011\right]$, together with two time indicators $\nu_{ijk}^{\left(HD\right)}=\left[1212311\right]$ and $\nu_{ijk}^{\left(DH\right)}=\left[11231\right]$. The time indicators numbered the intervals until a transition occurred (i.e. the elements in **f***_ijk _*until a 1 appears) or till the end of the sequence is reached. Since a cow could change states more than once during a lactation, the time indicators could start over several times. For the HD transitions, the counter started at 1 at the first measurement that is classified as healthy (most often the very first one) counting until a transition to a diseased state occurs. The counter started over again when or if the cow returned to a healthy state. For transitions in the other direction, the counter started when the cow developed the first case of classified mastitis and stopped when it returned to a healthy state. If the cow returned to a diseased state, the counter started over again. A cow without a case of classified mastitis did therefore not generate any data for transitions in the D to H direction. At the same time, the duration of a diseased state was usually much shorter than that of a healthy state. This resulted in a strongly reduced data-set for D to H transitions in comparison to the data in the H to D direction. To complement **f***_ijk _*and ***v**_ijk_*, three more sequences **k***_ijk_*, **s***_ijk _*and **h***_ijk _*were created, which contained indicators for cow, sire and herd. Each sequence was just a replicate of the same indicator duplicated as many times as the length of **f***_ijk _*and ***v**_ijk_*.

The expanded data material for the HD direction contained $f_{ijk}^{\left(HD\right)}$, $\nu_{ijk}^{\left(HD\right)}$, $k_{ijk}^{\left(HD\right)}$, $s_{ijk}^{\left(HD\right)}$ and $h_{ijk}^{\left(HD\right)}$ for all cows, while the data in the DH direction contained, $f_{ijk}^{\left(DH\right)}$, $\nu_{ijk}^{\left(DH\right)}$, $k_{ijk}^{\left(DH\right)}$, $s_{ijk}^{\left(DH\right)}$ and $h_{ijk}^{\left(DH\right)}$ for all cows that had at least one case of classified mastitis.

The transition probabilities in (2) can be expressed in terms of **f***_ijk_*. The probability of developing mastitis for cow *i *can be expressed as

(9) $$\pi_{ijk}^{\left(HD\right)}=P\left(f_{i\left(t+1\right)}^{\left(HD\right)}=1\left|f_{it}^{\left(HD\right)}=0\right)\right)$$

and was the same for cow *i *throughout the entire lactation, i.e. for all values of *t*, (t = 1,..., *T_i_*-1). The probability to recover was consequently expressed as

(10) $$\pi_{ijk}^{\left(DH\right)}=P\left(f_{i\left(t+1\right)}^{\left(DH\right)}=1\left|f_{it}^{\left(DH\right)}=0\right)\right).$$

### Analysis of simulation results

Breeding values were estimated in separate analyses for the two transitions directions (HD and DH). Correlations between true breeding values (TBV) and estimated breeding values (EBV), i.e. accuracy of selection, for both HD $\left(r_{BV}^{\left(HD\right)}\right)$ and DH $\left(r_{BV}^{\left(DH\right)}\right)$ directions, were calculated for all combinations of population sizes, mastitis frequencies, and genetic correlations (*r_G_*). In addition, correlations between EBV in the HD and DH directions were calculated for the same combinations in order to check if the negative, neutral and positive genetic correlations between mastitis and recovery liabilities in the simulated data are reproduced by the method.

## Results

As shown in Table 2 a larger daughter group size, higher mastitis frequency and a higher boundary level generated higher accuracies of EBV ($\left(r_{BV}^{\left(HD\right)}\right)$ and $\left(r_{BV}^{\left(DH\right)}\right)$). For mastitis liability, the method generated correlations between TBV and EBV that ranged from 0.53 to 0.83. For recovery liabilities, it was clear that the severely reduced dataset affected the estimates unfavorably. Because of the large number of cows without mastitis, the datasets were reduced to sizes between 1/6 and 2/5 of the original data. Despite this, the method managed to generate rather high accuracies even in the DH direction (0.25 to 0.62).

**Table 2:** Average correlations between true and estimated breeding values

	Daughters/sire	60				150			
	**Mastitis frequency (cases/lactation)**	**0.28** **Scenario 1**		**0.95** **Scenario 2**		**0.28** **Scenario 1**		**0.95** **Scenario 2**	
	**Transition direction**	**HD**	**DH**	**HD**	**DH**	**HD**	**DH**	**HD**	**DH**

*r_G _*= 0	*B*(*τ*) = 10 × *L*(*τ*)	**0.532**	**0.249**	**0.697**	**0.387**	**0.749**	**0.381**	**0.836**	**0.550**
		(0.012)	(0.018)	(0.012)	(0.021)	(0.009)	(0.016)	(0.005)	(0.013)
	Cows	24000	4149	24000	10151	60000	10374	60000	25450
	Herds	1200	1095	1200	1197	1200	1187	1200	1200
	
	*B*(*τ*) = 15 × *L*(*τ*)	**0.549**	**0.271**	**0.702**	**0.436**	**0.751**	**0.410**	**0.838**	**0.619**
		(0.007)	(0.013)	(0.012)	(0.018)	(0.009)	(0.012)	(0.005)	(0.006)
	Cows	24000	4128	24000	10139	60000	10328	60000	25411
	Herds	1200	1092	1200	1197	1200	1186	1200	1200
	
*r_G _*= 0.2	*B*(*τ*) = 10 × *L*(*τ*)	**0.589**	**0.268**	**0.684**	**0.407**	**0.760**	**0.386**	**0.834**	**0.533**
		0.0148	(0.015)	0.0108	(0.014)	(0.013)	(0.028)	(0.007)	(0.016)
	Cows	24000	4131	24000	10161	60000	10366	60000	25453
	Herds	1200	1094	1200	1198	1200	1184	1200	1200
	
	*B*(*τ*) = 15 × *L*(*τ*)	**0.592**	**0.301**	**0.690**	**0.467**	**0.766**	**0.436**	**0.837**	**0.612**
		(0.013)	(0.012)	(0.011)	(0.016)	(0.013)	(0.021)	(0.006)	(0.010)
	Cows	24000	4115	24000	10148	60000	10322	60000	25425
	Herds	1200	1093	1200	1197	1200	1183	1200	1200
	
*r_G _*= -0.2	*B*(*τ*) = 10 × *L*(*τ*)	**0.588**	**0.236**	**0.683**	**0.396**	**0.764**	**0.372**	**0.820**	**0.562**
		(0.017)	(0.019)	(0.013)	(0.021)	(0.011)	(0.021)	(0.015)	(0.012)
	Cows	24000	4136	24000	10189	60000	10423	60000	25428
	Herds	1200	1087	1200	1123	1200	1187	1200	1200
	
	*B*(*τ*) = 15 × *L*(*τ*)	**0.591**	**0.257**	**0.688**	**0.431**	**0.766**	**0.468**	**0.824**	**0.607**
		(0.016)	(0.019)	(0.011)	(0.013)	(0.010)	(0.045)	(0.014)	(0.011)
	Cows	24000	4118	24000	10176	60000	10408	60000	25396
	Herds	1200	1084	1200	1195	1200	1187	1200	1200

Correlations (in bold) between true and estimated breeding values in the healthy to diseased (HD) and diseased to healthy (DH) directions ($\left(r_{BV}^{\left(HD\right)}\right)$ and $\left(r_{BV}^{\left(DH\right)}\right)$) for different combinations of genetic correlation (*r_G_*), daughters per sire, mastitis frequency, and boundary level (*B*(*τ*)); the number of cows and herds used for each correlation are also reported; reported values in the table are the mean and standard errors (within parentheses) based on five replicates

There were no major differences in the correlations between TBV and EBV when comparing them for the different simulated values of the genetic correlations between mastitis and recovery liabilities (*r_G _*= -0.2, 0.2, and 0) (Table 2). Whether or not the different values of *r_G _*in the simulated data are reproduced in the calculated correlation between EBV in the HD and DH direction are reported in Table 3. Calculated correlations were rather scattered but with a pattern showing that the method manages to acknowledge the positive and zero simulated values of *r_G _*by generating calculated correlations of approximately the same magnitude as *r_G_*. However, the negative values of *r_G _*in the simulated data are not reproduced in the calculated correlations between the EBV in the two directions. Instead, these estimated correlations have values around zero.

**Table 3:** Average correlations between estimated breeding values in the HD and DH direction

Daughters/sire		60		150	
**Mastitis frequency (cases/lactation)**		**0.28** **Scenario 1**	**0.95** **Scenario 2**	**0.28** **Scenario 1**	**0.95** **Scenario 2**

*r_G _*= 0	*B*(*τ*) = 10 × *L*(*τ*)	**0.048** (0.027)	**0.060** (0.025)	**0.020** (0.018)	**0.003** (0.019)
	
	*B*(*τ*) = 15 × *L*(*τ*)	**0.074** (0.010)	**0.172** (0.025)	**0.066** (0.017)	**0.134** (0.016)

*r_G _*= 0.2	*B*(*τ*) = 10 × *L*(*τ*)	**0.125** (0.092)	**0.115** (0.005)	**0.056** (0.021)	**0.134** (0.032)
	
	*B*(*τ*) = 15 × *L*(*τ*)	**0.113** (0.026)	**0.167** (0.024)	**0.114** (0.019)	**0.270** (0.031)

*r_G _*= -0.2	*B*(*τ*) = 10 × *L*(*τ*)	**-0.005** (0.008)	**-0.003** (0.026)	**-0.074** (0.030)	**-0.068** (0.042)
	
	*B*(*τ*) = 15 × *L*(*τ*)	**0.046** (0.004)	**0.079** (0.016)	**-0.037** (0.022)	**0.041** (0.047)

Correlations (in bold) between estimated breeding values in the healthy to diseased direction and the diseased to healthy direction for different combinations of genetic correlation (*r_G_*), daughters per sire, mastitis frequency, and boundary level (*B*(*τ*)). Reported values in the table are the mean and standard errors (within parentheses) based on five replicates

## Discussion

### Model

Mastitis has been the focus of several research projects and is considered in breeding programs in many countries (e.g., [40]). However, in the genetic evaluation of udder health, only the mastitis liabilities are taken into account, leaving aside the recovery process.

Our main objective was to evaluate a new method that simultaneously models transitions to and from states of mastitis considering both the mastitis liability and the recovery process. For example, the method can distinguish between two cows, each with mastitis once during lactation but one showing fast recovery and the other suffering from a protracted infection. Naturally, our aim is to have as few mastitis infections as possible. However, mastitis is unavoidable and a relatively frequent problem among dairy cows. Thus, the capacity for fast recovery is also of interest.

The multilevel discrete time survival model is well suited to analyze the repeated nature of mastitis data. Green et al. [41] used a similar model to investigate how cow, farm and management factors during the dry period influence the incidence of clinical mastitis after calving. However, in their study, only the first case of mastitis was considered, leaving out repeated events in the analysis. The method's ability to take repeated mastitis cases within the same lactation into account was shown in our study through higher correlations between TBV and EBV for higher mastitis frequencies.

Besides the extra genetic information that can be captured by considering repeated cases, it has another beneficial aspect. A higher mastitis frequency reduces the proportion of falsely classified cases, which in turn gives more accurate EBVs. For example, a 10% mastitis misclassification rate among a group of 100 cows with 10 real cases leads to nine falsely classified individuals (10% of the 90 that do not have mastitis). This means 47% (9 of 19) of the mastitis classifications are false. The same misclassification rate among an equally large group, but with 30 real cases, results in seven falsely classified individuals (10% of the 70 that do not have mastitis). In this case, 19% (7 of 37) of the mastitis classifications are false. A higher classification error percentage for lower mastitis frequencies also appears if the misclassification goes in the other direction, i.e. mastitis cases are falsely classified as non-mastitis.

### Classification

Misclassifications are unavoidable when SCC is used as an indicator of mastitis. If the boundary between H and D is too low, high random fluctuations around "normal" SCC levels will lead to falsely classified cases of mastitis. There is a trade-off between classifying normal but elevated SCC values as infected if the level is too low and missing possible infections if the level is too high. The results in Table 2 show that the higher boundary level gives higher correlations between EBV and TBV. This is especially apparent for DH transitions, for which the reduced data was more sensitive to misclassifications than the complete data. Boundaries higher than 15×L(τ) were also tested (not reported here) but did not give an increase in accuracies. On the contrary, if the levels are too high, the results deteriorate.

In this simplified simulation study, the setting of the boundaries (and its consequences) are of course easier to determine than in a real-life dataset. However, the SCC response to a mastitis case was based on real-life observations and the boundaries should therefore be reasonably correct to use also in real-life data.

### Performance

The simulated data in this study was used previously to evaluate linear models (LM), threshold models (TM) and survival analysis (SA) [33]. Confirmed cases of mastitis were modeled either as a binary variable (separating absence of mastitis and 1 or more cases during lactation) or as time to first case. Correlations between TBV and EBV in that study were 0.53-0.60 and 0.70-0.76 for 60 and 150 daughters, respectively. The mastitis lactation percentage coincides with scenario 1 in this paper, i.e. the lower mastitis frequency. The corresponding correlations for scenario 1 of the transition method are 0.55-0.59 and 0.75-0.77 (Table 2), showing that the results for the transition method are well in line with those in [33]. Considering that the transition method analyzes SCC values as an indicator of mastitis, while the previous methods analyzed confirmed mastitis cases, the results are even more promising. When the mastitis frequency was higher, the transition method generated even higher correlations, i.e. up to 0.7 for 60 daughters and 0.84 for 150 daughters. A higher mastitis frequency leads to a larger number of lactations with more than one case. More individuals with repeated events makes the transition method even more favorable compared to the other three methods. These results show good prospects for second and higher lactations.

An additional advantage of the transition method is the information generated on the recovery process. These results are not comparable to the LM, TM, and SA methods, because of their one-way approach. However, survival analysis could be used in a similar manner as the transition method. By analyzing the time between infection and recovery, the results could be compared with the recovery results from the transition method. Nevertheless, traditional survival analysis does not take repeated cases into account, neither in the DH nor in the HD direction, which means that a cow can only develop mastitis once and recover once. However, there are a few attempts to analyze repeated cases, where one suggestion is the survival score model [42].

A positive genetic correlation between mastitis and recovery liabilities has two characteristics: cows that easily develop an infection also recover easily (Profile 1), while cows with high disease resistance have a longer recovery time (Profile 2). Cows with Profile 1 have a better chance to generate accurate EBV for their sires than cows with Profile 2 because cows with Profile 1 show their true capacity of moving between states without getting stuck in one of the two states. Cows with a high resistance to the disease, cannot show there aptitude for recovery, simply because they rarely develop mastitis. They are "stuck" in the healthy state.

A negative genetic correlation between mastitis and recovery liabilities is reflected by having cows with low mastitis resistance and long recovery time (Profile 3) and cows with high disease resistance and quick recovery (Profile 4). The inertia factor is even more apparent for data with a negative genetic correlation between mastitis and recovery liabilities, because it is then present for data of both characteristics i.e. Profile 3 and 4. Cows with Profile 3 do not show their inclination for repeated events because they easily get stuck in a diseased state. Among the cows in Profile 4, their inclination for fast recovery will not be evident because these cows hardly develop mastitis. The inertia factor is probably the major reason why the zero and positive genetic correlations are correctly estimated by the method while the negative genetic correlations are not.

Mastitis and recovery liabilities could be estimated jointly in a bivariate model with a prior on the correlation between the two liabilities. In this study, we decided against this for practical reasons and because of the few transitions that occur for the same cow (0.28 (0.95) mastitis cases per lactation), together with the inertia factor, could easily make the estimates of the correlations very imprecise. Even for the high frequency scenario, half of the cows had no mastitis and it is impossible (or at least very model dependent) to determine what the recovery rate is for cows that do not develop mastitis. Genetic correlation between mastitis and recovery liability could be estimated more accurately with multiple lactations per cow and/or from covariances on a sire level. Whether a genetic correlation exists between mastitis and recovery liability is yet to be investigated but the inertia factor should be recognized in such studies.

One should remember that the method used to generate the simulated data is not logistic-normal but uses a probit model. In spite of this, the logit analysis gave estimates close to the values used in data generation. This indicates that our method is not very sensitive to model specification errors. Use of a probit model for analysis is simpler than the logit model, in the sense that is does not require a MH step in the Gibbs sampler. However, the logistic model is less sensitive to outliers for explanatory variables. In practice, the generating mechanism behind the data is unknown, making it difficult to specify the correct model for data analysis. However, the two models usually give very similar results [43].

### Further developments

Different possibilities for developing and improving the method could be investigated. One possibility is to develop the method by adding a new state representing subclinical mastitis cases. We would then model transition probabilities between three possible states. The method could also be used to analyze repeated confirmed mastitis cases instead of SCC, excluding the need to classify cows based on SCC. However, complete data of confirmed or veterinary-treated cases are rather rare in field data but sometimes a combination of the two data types is available. The method could accommodate all available data in the genetic evaluation by combining SCC data with incomplete mastitis data. Generalizing the model to allow for time-dependent transition probabilities is another possibility which could be done by including a lactation stage effect in the analysis of the transitions.

Mastitis classification may be improved by going from a strict limit between states to a more flexible and realistic classification. Mixture models have been used successfully to classify mastitis types, e.g. [44,45], but not in combination with the transition probability model. Multivariate mixture models give the possibility to classify mastitis on the basis of more than one variable and to model overlapping groups, which may improve classification even further.

Associations between pathogen-specific mastitis and SCC pattern have been demonstrated [15,16]. Different patterns distinguish between long or short increase in SCC and also between lactations with or without recovery. Transition probabilities could be used to describe characteristic patterns of SCC and to identify pathogen-specific mastitis. The transition probability method may also be suitable for a wide range of other diseases for which individuals fall in and out of two or more states and the states are either confirmed or classified by one or more classification variables.

## Conclusions

This paper presents and evaluates a novel longitudinal model for genetic evaluation of mastitis. The model captures the dynamic nature of the disease by modeling mastitis liability and by including the recovery process and repeated cases into the analysis. Although a more complete evaluation of the method is necessary, especially on field data, the results point towards a significant gain when broadening the genetic evaluation of udder health to include the whole disease course.

## Competing interests

The authors declare that they have no competing interests.

## Authors' contributions

JF was involved in all steps of the study. Together with DT she developed the statistical method. She participated in the design of the study, simulated data, performed the statistical analysis, and drafted the manuscript. DT came up with the original statistical approach to tackle the needs and ambitions stated by ES. JIU participated in the design of the study and the data simulations. ES conceived the study and participated in its design and coordination and helped to draft the manuscript. All authors read and approved the final manuscript.
